# The Beat

**Published:** 2010-06

**Authors:** Erin E. Dooley

## EPA Proposes New Coal Ash Rules

Amid growing concerns about the health impacts of coal ash, the waste produced at coal-fired power plants, the U.S. EPA on 4 May 2010 issued a prepublication version of two options for the management and cleanup of such facilities.^1^ The agency is requesting public comments on two approaches to regulation

More stringent regulation as hazardous waste under Subtitle C of the Resource Recovery and Conservation Act (RCRA) would require the development of permit programs, allow for mandatory monitoring and federal enforcement, and more strictly control the use of impoundment liners and groundwater monitoring. Regulation as nonhazardous solid waste under Subtitle D puts more control in the hands of individual states. The EPA also seeks comment on potential refinements to the list of beneficial uses allowed. The proposals will be subject to the EPA’s usual 90-day period for public comment upon publication in the *Federal Register*; at press time, the proposals had not yet been published.^2^

## Study Shines Light on the Molecular Structure of Fluorescent Proteins

Researchers have determined the crystal structures of two key fluorescent proteins, one blue and one red, that can be used to highlight molecules in cells being studied.^3^ This discovery could allow scientists to more systematically design novel fluorescent proteins for pinpointing structures and processes in living cells, with more choices of color allowing the simultaneous observation of more individual processes within cells.

**Figure:**
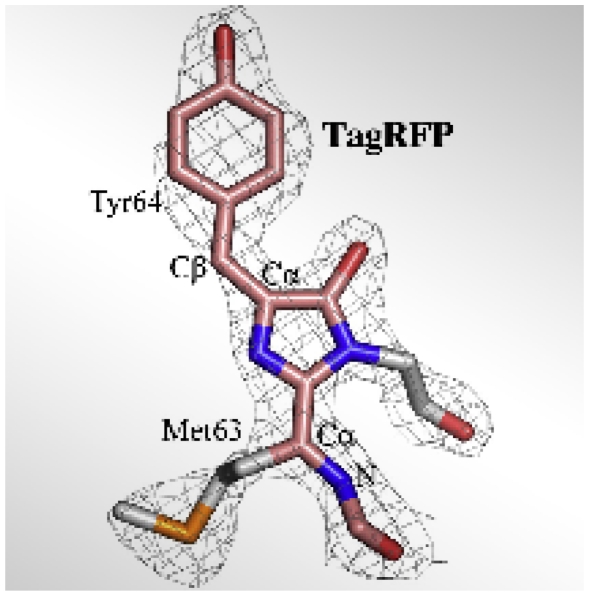
The backbone chain that gives the TagRFP red fluorescent protein its color.

## Stream Quality May Predict Human Health

An interdisciplinary study by Nathaniel Hitt and Michael Hendryx looked at the relationship between human cancer mortality in West Virginia and the ecologic integrity of nearby stream ecosystems.^4^ They found that people living near the most biologically impoverished streams—as reflected by the numbers and proportions of bottom-dwelling macroinvertebrates living there—had the highest cancer rates, even after adjusting for known risk factors such as smoking, income level, and urbanization. Spatial analyses also revealed more cancers in areas where more coal mining occurred. Their results suggested that proximity to coal surface mines can potentially lead to adverse effects in both aquatic and human communities.

## Chesapeake Bay Enforcement Actions Now Online

In April 2010, the EPA announced a new effort to provide public online access to its work in enforcing federal pollution laws in the Chesapeake Bay region.^5^ The move includes a new focus on targeting the sources responsible for contributing the greatest amounts of nitrogen, phosphorus, and sediment to the watershed. Last year is the first time the agency has compiled enforcement statistics for the Chesapeake Bay region. According to the EPA, actions pursued since 2009 will keep more than 15 million pounds of nitrogen oxides out of the bay airshed along with 2,100 pounds of nitrogen and phosphorus and 82 million pounds of sediment out of the bay watershed once all the required controls are put in place.

**Figure:**
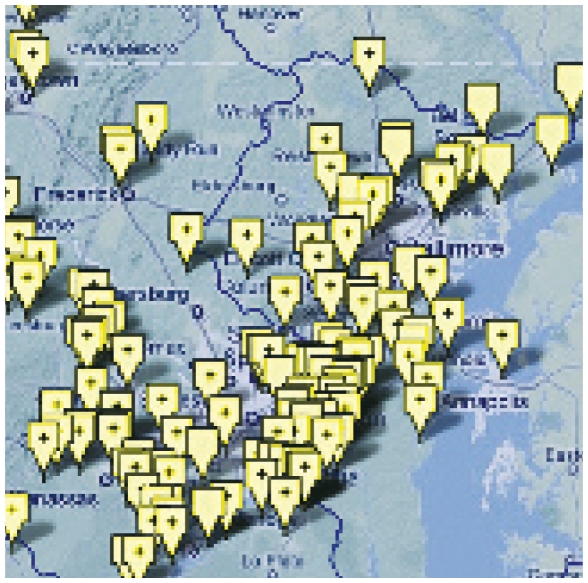
Yellow flags indicate sites of enforcement actions taken in the Chesapeake Bay airshed/watershed.

## NRC Reports on Ocean Acidification

A recent National Research Council report states the increasing acidification of the world’s oceans, a result of CO_2_ uptake, will continue to worsen if anthropogenic CO_2_ emissions are not curbed.^6^ Recent legislation calls for establishment of a national program to study and respond to the effects of ocean acidification. To date, the ocean has absorbed about a third of the anthropogenic CO_2_ released as a result of human activities. The report describes six elements the authors consider key to a successful National Ocean Acidification Program.

## COSMOS Cell Phone Study Launched

More than 250,000 people are expected to be enrolled in a new 30-year European study, COSMOS,^7^ the largest of its kind to date, on the effects of cell phone use on human health. Although most of the research to date has found little link between cell phone use and health effects, there is some concern that cell phones have not been in common use long enough for such effects to be determined through studies. The new study will look not only at cancer, but also at stroke, heart disease, neurodegenerative diseases, headaches, sleep disorders, and tinnitus.
